# Using Network Pharmacology to Explore the Mechanism of Panax notoginseng in the Treatment of Myocardial Fibrosis

**DOI:** 10.1155/2022/8895950

**Published:** 2022-03-25

**Authors:** Jingxue Han, Jingyi Hou, Yu Liu, Peng Liu, Tingting Zhao, Xinwei Wang

**Affiliations:** ^1^Beijing Key Lab for Immune-Mediated Inflammatory Diseases, Institute of Clinical Medical Sciences, China-Japan Friendship Hospital, Beijing 100029, China; ^2^Heilongjiang Academy of Chinese Medical Sciences, Harbin 150036, China; ^3^School of Chinese Materia Medica, Beijing University of Chinese Medicine, Beijing 100029, China; ^4^Shunyi Hospital, Beijing Traditional Chinese Medicine Hospital, Beijing 101300, China

## Abstract

**Objective:**

The mechanism of Panax notoginseng in treating myocardial fibrosis (MF) was investigated using network pharmacology.

**Methods:**

Effective ingredients and potential targets of Panax notoginseng were screened in relevant databases to construct a compound-target network. Targets of MF were then screened to select common targets and construct a protein-protein interaction network. This was followed by Gene Ontology and pathway enrichment analyses. Molecular docking then verified the results of network analysis.

**Results:**

A total of 14 effective ingredients and 119 potential targets for MF were predicted. Quercetin, beta-sitosterol, and gossypetin were speculated to be the main active ingredients. The mechanism of action may be related to AGE-RAGE, proteoglycans, and IL-17 signaling pathways. Five key targets (IL6, ALB, AKT1, TNF, and VEGFA) may be involved in the treatment of MF using Panax notoginseng.

**Conclusions:**

This study embodies the complex network relationship of multicomponents, multitargets, and multipathways of Panax notoginseng in treating MF and provides a novel method for further research on this herb's mechanism.

## 1. Introduction

Cardiovascular disease (CVD) is the leading cause of mortality worldwide, and it is estimated that CVD will result in more than 23 million deaths by 2030 [1]. Diabetes mellitus is widely known as a major risk factor for CVD. When glucose is not well-controlled in either type 1 or 2 diabetes, vascular and nerve damage can occur over time [2–4]. Damage to the heart vessels can lead to CVD. Myocardial fibrosis (MF) is a pathophysiologic process of many cardiovascular diseases [5, 6]. MF is the result of persistent and/or repeated damage and stress from various causes. These can include myocardial ischemia and hypoxia due to coronary atherosclerotic stenosis resulting from diabetes mellitus [7, 8]. Drugs such as angiotensin-converting enzyme inhibitors, beta-blockers, statins, and agents that target fibrosis have some beneficial effects. However, they cannot prevent the progression of MF and have certain side effects [9]. Thus, alternative therapies such as traditional Chinese medicine may be a treatment option for MF with fewer side effects and lower cost.

Panax notoginseng (Burk.) F.H. Chen (notoginseng) is an herb commonly used in Chinese medicine. Its traditional application is to promote blood circulation and dispel blood stasis. Records of Panax notoginseng date to the *Compendium of Materia Medica* (*Ben Cao Gang Mu*) compiled by Li Shizhen in the Ming dynasty. Modern research has revealed that the main components of Panax notoginseng include saponins, volatile oils, flavonoids, and polysaccharides. Its pharmacologic effects are mainly reflected in its actions on the circulatory and cerebrovascular systems. In murine experiments, Panax notoginseng has been found to have a therapeutic effect on MF [10–12]. However, its mechanism of action remains unclear.

**Figure 1:**
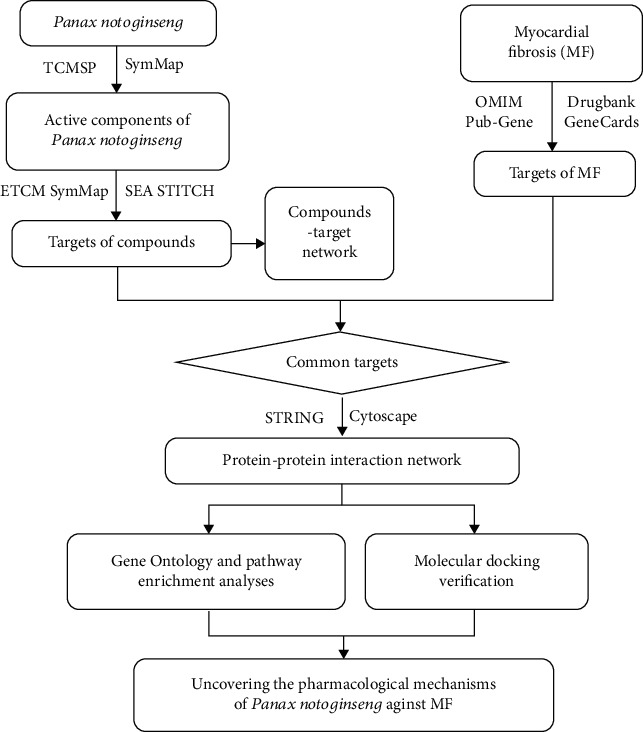
Flow diagram of the pharmacology-based study of Panax notoginseng used in treating MF.

Network pharmacology is a systematic research methodology that combines laboratory and clinical inquiries with data processing to guide drug discovery and development. It is an effective method for studying the complex relationship between Chinese herbal medicines and diseases [13]. This current study uses network pharmacology methods to elucidate the potential mechanism of Panax notoginseng in the treatment of MF and provides a basis for subsequent pharmacologic experimental studies (Figure 1).

## 2. Materials and Methods

### 2.1. Screening of Compound Components

The keywords “Panax notoginseng” were used to retrieve the compound components in the SymMap database (http://www.symmap.org) and in the Traditional Chinese Medicine Systems Pharmacology Database and Analysis Platform (TCMSP) database (https://tcmspw.com/tcmsp.php). The screening criteria were oral bioavailability (OB) ≥ 30% and drug‐like (DL) ≥ 0.18.

### 2.2. Construction of the Component-Target Network

The targets of the compounds were searched through the Encyclopedia of Traditional Chinese Medicine (ETCM) database (http://www.nrc.ac.cn:9090/ETCM/index.php/Home/Index/index.html), the SymMap database, the Similarity Ensemble Approach (SEA) database (http://sea.bkslab.org), and the STITCH database (http://stitch.embl.de). The UniProt ID of the target was then searched through the Universal Protein Resource (UniProt) database (https://www.uniprot.org), with the species defined as “Homo sapiens.” All gene names were assigned their official gene symbol. Then, targets that did not meet the screening criteria were eliminated. Next, the network mapping software Cytoscape 3.8.0 (http://www.cytoscape.org) was used to construct networks for the compounds and their targets. In the network, a node represents a target, gene, molecule, or protein, and the connections between nodes represent the interactions between the targets, genes, molecules, or proteins. The “degree” value of a node represents the number of connections between the nodes in the network; the larger the value, the more likely the target is to become the key target of compounds.

### 2.3. Acquisition of Disease Targets

The keyword “myocardial fibrosis” was searched in the Online Mendelian Inheritance in Man (OMIM) database (https://omim.org), GeneCards database (https://www.genecards.org), Drugbank database (https://www.drugbank.ca), and Pub-Gene database (https://www.ncbi.nlm.nih.gov/pubmed) to obtain the disease targets.

### 2.4. Construction and Analysis of the Protein-Protein Interaction (PPI) Network

The potential targets of the retrieved compounds and disease targets were intersected, and the overlapping targets were selected and imported into the STRING database (https://string-db.org) to obtain the protein interaction relationship. The results were then imported into Cytoscape 3.8.0 software to construct and analyze the interaction network.

### 2.5. Screening of Core Clusters and Key Targets

Cytoscape plugin MCODE was applied for cluster analysis, and the filter conditions were set as degree cutoff: 2, *k*-core: 2 to select the core cluster with the closest relationship in the network. Then, the plugin CytoHubba was applied to analyze the PPI network and core cluster to obtain the network topology parameters. The targets shared by both the PPI network and core cluster with a high degree were selected as the key targets, which were retrieved in the DisGeNET database (http://www.disgenet.org/search) to obtain the protein class of key targets.

### 2.6. Gene Ontology and Pathway Enrichment Analyses

The Gene Ontology (GO) database (http://geneontology.org) includes various functions of genes including biologic process (BP), molecular function (MF), and cellular component (CC) and can be applied to the analysis of potential biologic molecular mechanisms. The KEGG database (https://www.kegg.jp) is used to identify biologic functions and candidate targets. In this study, ClusterProfiler (https://bioconductor.org/packages/release/clusterProfiler.html) in R package was applied to GO functional annotation and KEGG pathway analysis, and the enrichment analysis results were visualized.

### 2.7. Molecular Docking Verification

The Ligand Docking module in Schrödinger software was used to verify the reliability of the results, and the binding activity of the compound to the key targets was evaluated by the docking score. The structures of all the compounds were downloaded from the PubChem database (https://pubchem.ncbi.nlm.nih.gov/), and the three-dimensional structures of the key targets were downloaded from the Protein Database (PDB) (http://www.rcsb.org/pdb/home/home.do). The higher the absolute value of the docking score, the stronger the binding ability of small molecules to protease targets.

## 3. Results and Analysis

### 3.1. Compound Screening

Ten compounds were screened through the SymMap and TCMSP databases. The OB and DL values of notoginsenoside R1, ginsenoside Rg1, ginsenoside Rb1, and ginsenoside Rb2 were smaller than the screening criteria and were deleted by the system. However, through searching the literature, we found that these compounds are related to myocardial fibrosis and diabetes and thus included the compounds [11, 14–16]. Therefore, a total of 14 compounds were eventually contained in the follow-up study (Table 1).

### 3.2. Target Prediction and Network Analysis of Compounds

Potential targets of compounds through ETCM, SymMap, SEA, and STITCH databases were searched, and 829 targets of Panax notoginseng were obtained after deleting duplicates.

**Figure 2:**
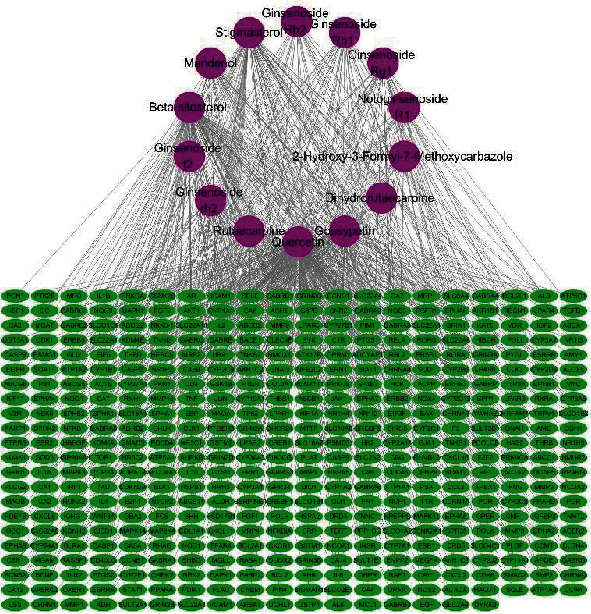
Effective component-target network. Violet nodes represent compounds of Panax notoginseng, and green nodes represent predicted targets.

Using Cytoscape 3.8.0, we constructed a network relationship among compounds and predicted targets (Figure 2). The resulting network included 451 nodes and 829 interaction edges. The degree values of compounds in the compound-target network were then obtained (Table 2). Quercetin has 238 potential targets, followed by beta-sitosterol with 121, gossypetin with 102, and stigmasterol with 96. These higher-degree compounds are likely to be involved in treatment of MF by Panax notoginseng.

### 3.3. Results of Disease Target Retrieval

With “myocardial fibrosis” as the keyword, a combined total of 601 myocardial fibrosis disease targets were found in the OMIM, Pub-Gene, Drugbank, and GeneCards databases after deleting duplicates.

### 3.4. Screening of Drug-Disease Targets

The intersections of potential targets of Panax notoginseng and disease targets resulted in 119 potential treatment targets for MF.

### 3.5. PPI Network of Panax notoginseng in the Treatment of MF and Key Target Analysis

**Figure 3:**
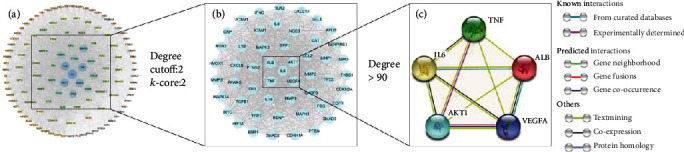
Network diagram of the PPI network, core clusters, and key targets: (a) PPI network; (b) core clusters; (c) key targets.

The PPI network was mapped using common potential targets of Panax notoginseng and MF, consisting of 119 nodes and 2597 interaction edges (Figure 3(a)). The CytoHubba plug-in was used to analyze the PPI network to obtain core clusters (Figure 3(b)) and key targets (degree > 90). The following are the five targets with the largest degree value: interleukin 6 (IL6), albumin (ALB), AKT serine/threonine kinase 1 (AKT1), tumor necrosis factor (TNF), and vascular endothelial growth factor A (VEGFA), whose protein class involves transfer/carrier protein, calcium-binding protein, kinase, transferase, and signaling molecule (Table 2). The network of key targets was constructed based on the STRING database (Figure 3(c)). In the network, the key targets interacted with each other through known (from curated databases and experimentally determined), predicted (gene neighborhood, gene fusions, and gene cooccurrence), and other (text mining, coexpression, and protein homology) interactions.

### 3.6. GO and KEGG Enrichment Analysis

**Figure 4:**
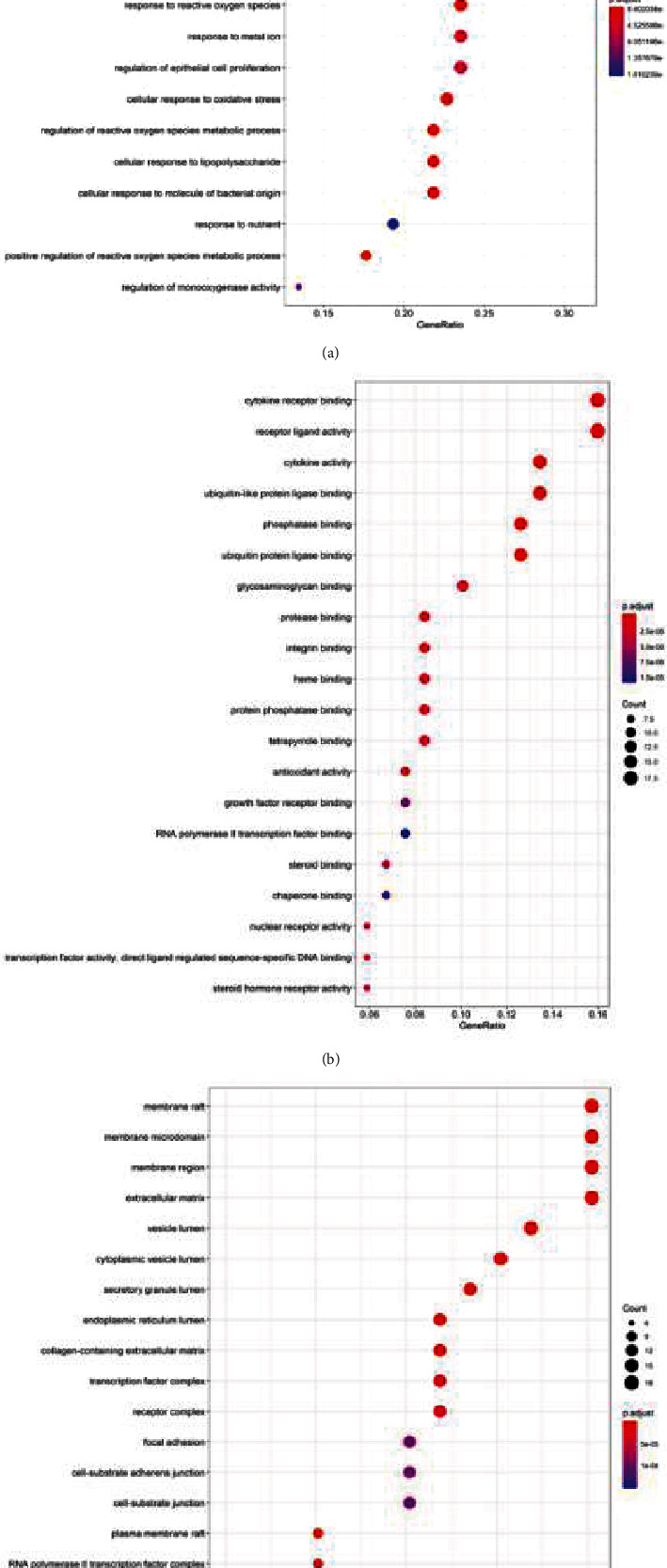
GO function enrichment analysis of potential targets from active ingredients of Panax notoginseng: (a) biological process; (b) molecular function; (c) cellular component.

GO functional annotation and KEGG pathway analysis were performed on 119 targets of the PPI network. The top 20 were then visualized as bubble charts (Figure 4). In the biological process, Panax notoginseng has great influence on nutrient levels, lipopolysaccharide, and molecule of bacterial origin (Figure 4(a)). At the molecular level, the function of drug components of Panax notoginseng is mainly related to cytokine receptor binding, receptor ligand activity, and cytokine activity (Figure 4(b)). Targets in the cellular components are closely related to membrane raft, membrane microdomain, and membrane region (Figure 4(c)).

**Figure 5:**
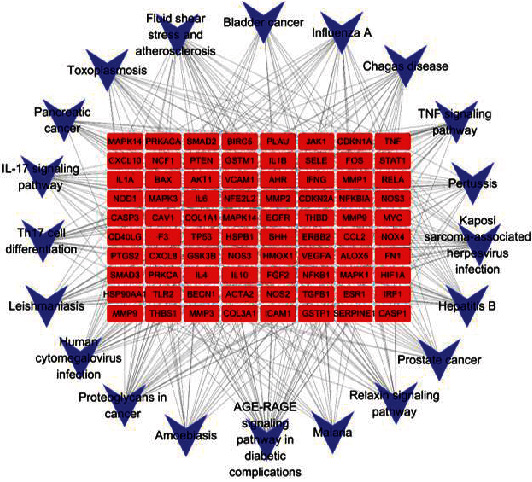
Target-KEGG pathway network. Blue nodes represent 20 KEGG pathways, and red nodes represent common targets.

A total of 230 enrichment results were obtained by KEGG pathway analysis. The first 20 pathways were screened according to adjusted *p* < 0.05 (Figure 5) and consisted of 101 nodes and 419 interaction edges, which mainly involve signaling pathways such as the advanced glycation end products-receptor for advanced glycation end product (AGE-RAGE) signaling pathway in diabetic complications, proteoglycans in cancer, fluid shear stress and atherosclerosis, and interleukin-17 (IL-17) signaling pathway, thus indicating that the effective components of Panax notoginseng might treat MF by acting on these pathways.

### 3.7. Verification of Results by Molecular Docking

**Figure 6:**
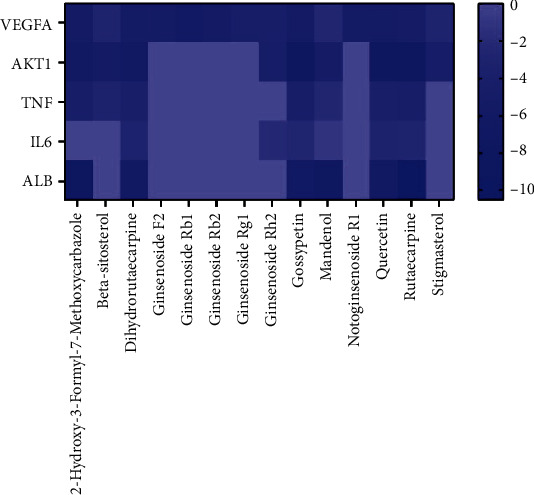
Heat maps of the docking scores of key targets combining with bioactive compounds in Panax notoginseng.

The key targets IL6, ALB, AKT1, TNF, and VEGFA were used for molecular docking with the effective compounds of Panax notoginseng, and a heat map was drawn based on the results (Figure 6). All bioactive components of Panax notoginseng had good binding with key targets, suggesting that Panax notoginseng has a strong tendency as a therapeutic strategy for MF via these key targets.

**Figure 7:**
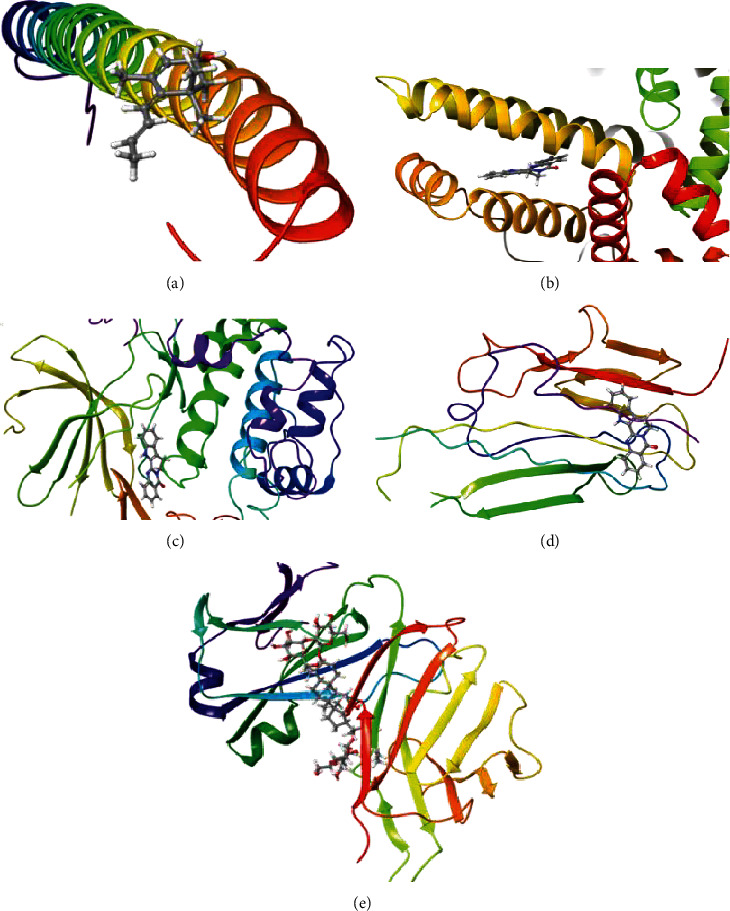
Molecular docking simulation of bioactive compound-key target: (a) IL6-dihydrorutaecarpine; (b) ALB-rutaecarpine; (c) AKT1-rutaecarpine; (d) TNF-rutaecarpine; (e) VEGFA-ginsenoside Rb1.

Results showed that rutaecarpine has a strong binding ability with ALB (docking score = −10.526), AKT1 (docking score = −8.277), and TNF (docking score = −4.689) (Figure 7). Dihydrorutaecarpine has a strong binding ability to IL6 (docking score = −3.345) and ginsenoside Rb1 with VEGFA (docking score = −6.188).

## 4. Discussion

This study used network pharmacology to systematically analyze the mechanism of action of Panax notoginseng in the treatment of myocardial fibrosis (MF). The resulting PPI network has 119 targets, accounting for one-third of the target number of Panax notoginseng. Five key targets, IL6, ALB, AKT1, TNF, and VEGFA, have high network value. Thus, we speculate that the effective components of Panax notoginseng may have pharmacologic activities in the treatment of MF through these targets.

Fibrosis is the final stage of a chronic inflammatory response, which can be caused by many factors. IL6 is a potent proinflammatory cytokine involved in MF [17]. Fibroblasts maintain this potential pathogenic change by regulating the production of IL6. Overexpression of IL6 is sufficient to induce myofibroblast proliferation, differentiation, and fibrosis. IL6 is involved in ischemic myocardial remodeling by upregulating the TGF-*β*1 signaling pathway [18–20]. TNF-*α* is also a proinflammatory cytokine with a wide range of biologic effects and is involved in the pathophysiology of various cardiovascular diseases. Low-level expression of TNF-*α* in normal myocardium has a protective effect on myocardial cells. However, its increased expression can cause myocardial fibroblast proliferation, myocardial cell death, systolic dysfunction, cardiac fibrosis, and ventricular remodeling [21–23].

In this network pharmacology study, we found that ALB, VEGFA, and AKT1 are also involved in MF. Jäntti et al. found a close relationship between the ALB level and cardiac function. When the plasma ALB level of hemodialysis patients was increased, cardiac function of patients improved, thus effectively raising their quality of life [24]. Studies have found that VEGFA can induce angiogenesis, and in infarcted hearts, VEGFA-mediated cardiac stem cell engraftment resulted in a reduction in fibrosis [25, 26]. The Akt signaling pathway is involved in cardiac hypertrophy and remodeling. Short- and medium-term overexpression of AKT1 leads to physiologic hypertrophy, but long-term activation of AKT1 can lead to pathologic hypertrophy, such as systolic dysfunction [27]. Knockdown of AKT1 in macrophages can reduce transdifferentiation of fibroblasts, suggesting that AKT1, as an important signaling molecule, may regulate fibroblast transdifferentiation by promoting an inflammatory reaction [28].

Through this network pharmacology study, we found that Panax notoginseng in treating MF mainly involves the AGE-RAGE signaling pathway in diabetic complications, proteoglycans in cancer, and IL-17 signaling pathways. Cardiovascular complications are the leading cause of death in diabetic patients. The AGE-RAGE signaling pathway of myocardial fibrosis in diabetic complications has been widely studied. It regulates the pathogenesis of cardiovascular disease and promotes increased collagen deposition leading to tissue fibrosis [29, 30]. Therefore, targeting the AGE-RAGE signaling pathway is a potential therapeutic strategy for ameliorating CVDs in diabetes.

Recent studies have shown that proteoglycans are promising diagnostic biomarkers for cardiac fibrosis and may provide new treatment strategies for heart disease [31]. Proteoglycans are a nonstructural component of the extracellular matrix and regulate many aspects of the immune response [32, 33]. Decorin, a well-investigated proteoglycan, inhibits both bioactivity and gene expression of TGF-*β*, a powerful fibrogenic factor [34]. Furthermore, decorin gene transfer significantly attenuates interstitial fibrosis and cardiac hypertrophy [35]. In various forms of cardiac fibrosis, the expression of the four-membered family of transmembrane proteoglycans, syndecan-1 to syndecan-4, is upregulated in response to proinflammatory stimuli and regulating fibrosis [36].

IL-17 has also been found to be involved in MF. In diabetic mice, IL-17 can reduce MF and improve cardiac function by inhibiting long-term noncoding RNA-AK081284 [37]. Studies have found that IL-17 contributes to MF through the protein kinase C (PKC) *β*/Erk1/2/NF-*κ*B (nuclear factor *κ*appa B) pathway [38, 39]. Thus, it can be inferred that AGE-RAGE, proteoglycans, IL-17, and other signaling pathways appear to be closely related to the mechanism of Panax notoginseng in the treatment of MF.

We found that rutaecarpine and ginsenoside Rb1 are the prime binding compounds to the key targets through molecular docking. Rutaecarpine exhibits a number of pharmacologic effects on the cardiovascular system including cardiac protective, vasodilator, and antiatherosclerotic activities [40, 41]. Rutaecarpine has been found to significantly improve cardiac function and decrease the content of TNF-*α* in myocardial tissues [42]. Ginsenoside Rb1, an active saponin of Panax notoginseng, has anti-inflammatory and antioxidative functions. It decreases the heart rate, improves cardiac function, and attenuates histologic changes induced by heart failure. Furthermore, ginsenoside Rb1 has also been shown to protect cardiomyocytes by targeting microRNA-21 and reverse the imbalance between apoptosis and autophagy in atherosclerosis [43–45]. While research on the above compounds has revealed their potential effects on the heart muscle, the specific mechanisms of their antimyocardial fibrotic actions remain unclear and need further study.

## 5. Conclusion

Panax notoginseng has a wide range of clinical applications in treating MF, but there are few reports on its pharmacologic activities. In this network pharmacology study, a multicomponent, multitarget, and multipathway treatment of MF using Panax notoginseng was established and provides a theoretical basis for clinical treatment of MF. However, the results of this study are based on data analysis and have only a certain predictive effect, which needs to be verified by further in vitro and in vivo experiments.

## Figures and Tables

**Table 1:** Potential effective compounds of Panax notoginseng.

	Compound	OB%	DL	Degree	Structure
1	Quercetin	46.43	0.28	238	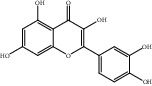
2	Beta-sitosterol	36.91	0.75	121	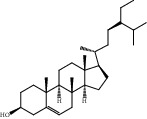
3	Gossypetin	35	0.31	102	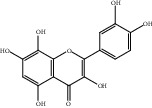
4	Stigmasterol	43.83	0.76	96	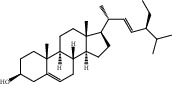
5	Ginsenoside Rg1	9.03	0.28	46	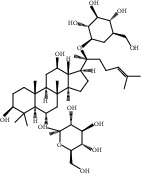
6	Ginsenoside Rh2	36.32	0.56	41	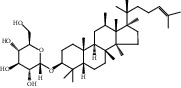
7	Mandenol	42	0.19	40	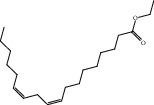
8	Ginsenoside Rb1	6.24	0.04	38	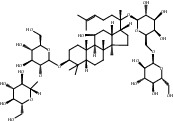
9	Rutaecarpine	40.3	0.6	36	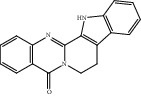
10	Ginsenoside Rb2	6.02	0.04	28	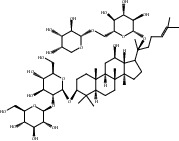
11	Notoginsenoside R1	4.27	0.13	26	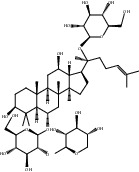
12	2-Hydroxy-3-formyl-7-methoxycarbazole	83.08	0.18	10	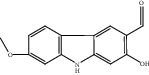
13	Dihydrorutaecarpine	42.27	0.6	4	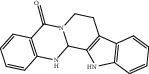
14	Ginsenoside F2	36.43	0.25	3	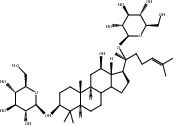

**Table 2:** Protein classes of key targets.

Gene name	Target	UniProt ID	Protein class	Degree
IL6	Interleukin 6	P05231	None	106
ALB	Albumin	P02768	Transfer/carrier protein	100
AKT1	AKT serine/threonine kinase 1	P31749	Calcium-binding protein; kinase; transfer/carrier protein; transferase	98
TNF	Tumor necrosis factor	P01375	Signaling molecule	98
VEGFA	Vascular endothelial growth factor A	P15692	Signaling molecule	95

## Data Availability

The data used to support the findings of the study are included within the article.
